# Computational modelling reveals distinct patterns of cognitive and physical motivation in elite athletes

**DOI:** 10.1038/s41598-018-30220-3

**Published:** 2018-08-08

**Authors:** Trevor T.-J. Chong, Matthew A. J. Apps, Kathrin Giehl, Stephanie Hall, Callum H. Clifton, Masud Husain

**Affiliations:** 1 Monash Institute of Cognitive and Clinical Neurosciences, Monash University, Victoria, 3800 Australia; 2 Department of Experimental Psychology, University of Oxford, Oxford, OX1 3UD United Kingdom; 3 Nuffield Department of Clinical Neurosciences, John Radcliffe Hospital, Oxford, OX3 9DU United Kingdom; 4 Department of Nuclear Medicine, University of Cologne, 50937 Cologne, Germany

## Abstract

Effort can be perceived both cognitively and physically, but the computational mechanisms underlying the motivation to invest effort in each domain remain unclear. In particular, it is unknown whether intensive physical training is associated with higher motivation specific to that domain, or whether it is accompanied by corresponding changes in cognitive motivation. Here, we tested a group of elite Oxford University rowers, and compared their behaviour to matched non-athletic controls. We trained participants on two tasks involving cognitive or physical effort. They then decided between a baseline low level of effort for low reward, versus higher levels of effort for higher rewards. Separate choices were made for the cognitive and physical tasks, which allowed us to computationally model motivation in each domain independently. As expected, athletes were willing to exert greater amounts of physical effort than non-athletes. Critically, however, the nature of cognitive effort-based decisions was different between groups, with a concave pattern of effort discounting for athletes but a convex pattern for non-athletes. These data suggest that the greater physical drive in athletes is accompanied by fundamentally different patterns of cognitive effort discounting, and suggests a complex relationship between motivation in the two domains.

## Introduction

The motivation to overcome effortful costs in pursuit of rewards is fundamental to everyday life. Students must decide how much cognitive effort to put into studying for an exam. Athletes must decide how much physical training to endure to win their next meet. Recent research has focused on understanding the cost-benefit trade-offs that are inherent to motivated behaviour^1–3^. These studies have emerged from a rich literature on non-human animals, which has traditionally focused on the willingness to pursue rewards associated with physical effort costs^4^. Importantly, however, effort can be experienced, not only in the physical domain, but cognitively as well^5–7^. A key question that remains is to what extent the mechanisms underlying motivation in one domain are generalisable to those in an alternate domain. Are individuals who are more physically motivated necessarily also more cognitively motivated?

Few studies have compared motivation across the cognitive and physical domains, and it therefore remains unclear whether high levels of motivation in one domain are necessarily accompanied by high motivation in the other. Recently, we used a neuroeconomic approach to determine the degree to which individuals are willing to invest cognitive or physical effort in return for reward^8^. This approach was based on the principle that effort is aversive^9^, and that rewards are devalued by the effort associated with acquiring them (‘effort discounting’)^10,11^. Cognitive and physical effort discounting recruited a largely overlapping network of domain-general regions, including the dorsomedial prefrontal cortex and dorsal anterior cingulate cortex^8^. This general finding is broadly consistent with the only other human neuroimaging study on cognitive and physical motivation, which also provided evidence for a domain-general, common network of brain regions involved in motivation^12^.

In addition to a domain-general network, there is also emerging evidence that rewards are discounted in distinct patterns across different domains of effort. In rodent studies, cognitive and physical motivation involve dissociable neurobiological substrates, with the amygdala playing a distinct role in cognitive effort discounting^13,14^. The distinct nature of cognitive and physical motivation was also evident in our recent human study, which showed that effort discounting in the two domains were described by separate computational functions. Specifically, cognitive effort discounting was best modelled as a convex (hyperbolic) function, in which changes at the lower levels of effort resulted in greater reward devaluation than changes at higher levels. In contrast, physical effort discounting was best modelled as a concave (parabolic) function, which describes the opposite pattern^15–20^. In keeping with the animal literature, the imaging data from this study also revealed dissociable substrates for cognitive and physical motivation, with the amygdala being uniquely sensitive to cognitive effort discounting^8^.

Here, we ask whether high motivation in one domain of effort (e.g., physical) is necessarily accompanied by high motivation in the other (e.g., cognitive)? One population that offers a unique insight into this issue are elite athletes. Elite athletes undertake frequent, intense, physical training sessions, with the goal of excelling at their sport^21–23^. Indeed, one of the most significant factors that affects sporting performance is the perceived physical demand of a task: as effort ratings increase, athletic performance declines^24^. Athletes are specifically trained to overcome these increases in perceived physical demand in pursuit of reward. Many studies in sports psychology have examined the relationship between motivation and performance^25^, and more recently some have proposed ‘psychobiological models’ of sporting performance that incorporate effort-based decisions^26,27^. To our knowledge, however, no study has sought to define the computational mechanisms underlying motivation and effort-based choice in athletes, either within or across the physical and cognitive domains.

In this study, we tested a group of elite Oxford University rowers, and compared their performance to that of age- and education-matched non-athletes. We used a computational approach to model participants’ responses, by calculating the ‘subjective value’ (SV) of engaging in an effortful action on every trial. By having participants make separate decisions for cognitive and physical effort, we were able to determine how effort discounting mechanisms differed between the physical and cognitive domains, and between the athlete and non-athlete groups.

## Results

### Participants

We tested 20 elite rowers, and 20 age- and education-matched non-athletic controls (Table 1). All participants were undergraduate or graduate students at the University of Oxford. Athletes were elite rowers competing at university or national level, and were recruited from the top crews of the University of Oxford rowing squads (the Oxford University Boat Club, Women’s Boat Club, Lightweight Rowing Club, and Women’s Lightweight Rowing Club). Each squad trains twice a day, six days per week, with the rowers in our study engaging in an average of 20.8 ± 0.4 hours of training per week over the 2.3 ± 0.5 years they had been in their respective squads. These characteristics are consistent with previous studies that have defined ‘elite‘/’expert’ athletes based on their level of competition^28,29^, experience^21,23^, and duration^30,31^ and frequency^32–34^ of training. Non-athletes had no history of prior competitive athletic experience. No participant had any neurological or psychiatric comorbidities. Participants were excluded if their reinforcement rates in the physical and/or cognitive effort tasks were <80% (two non-athletes, not included in the final sample of *N* = 20 + 20). This study was approved by the University of Oxford’s Central University Research Ethics Committee (MSD-IDREC-C1-2014-037), and was conducted in accordance with local guidelines. All participants gave written, informed consent for their participation.

**Table 1:** Participant demographics (mean ± 1 standard error).

	Athletes **(***n* = 20**)**	Non-Athletes**(***n* = 20**)**	Group Difference
Age	22.7 ± 0.62	22.8 ± 0.51	*t*(38) = 1.13, *p* = 0.27
Education (years)	14.6 ± 0.43	15.6 ± 0.44	*t*(38) = 1.70, *p* = 0.10
Gender (M:F)	7:13	10:10	χ^2^(1,40) = 0.41, *p* = 0.52
Duration of Training (hours/week)	**20**.**8** ± **0**.**4**	**0** ± **0**	***t*(38) = 57.5**, ***p < 0.001***
Years in Squad	**2**.**3** ± **0**.**5**	**0** ± **0**	***t*(38) = 4.87**, ***p < 0.001***

Significant group differences are indicated in bold.

### Behavioural Results

**Figure 1:**
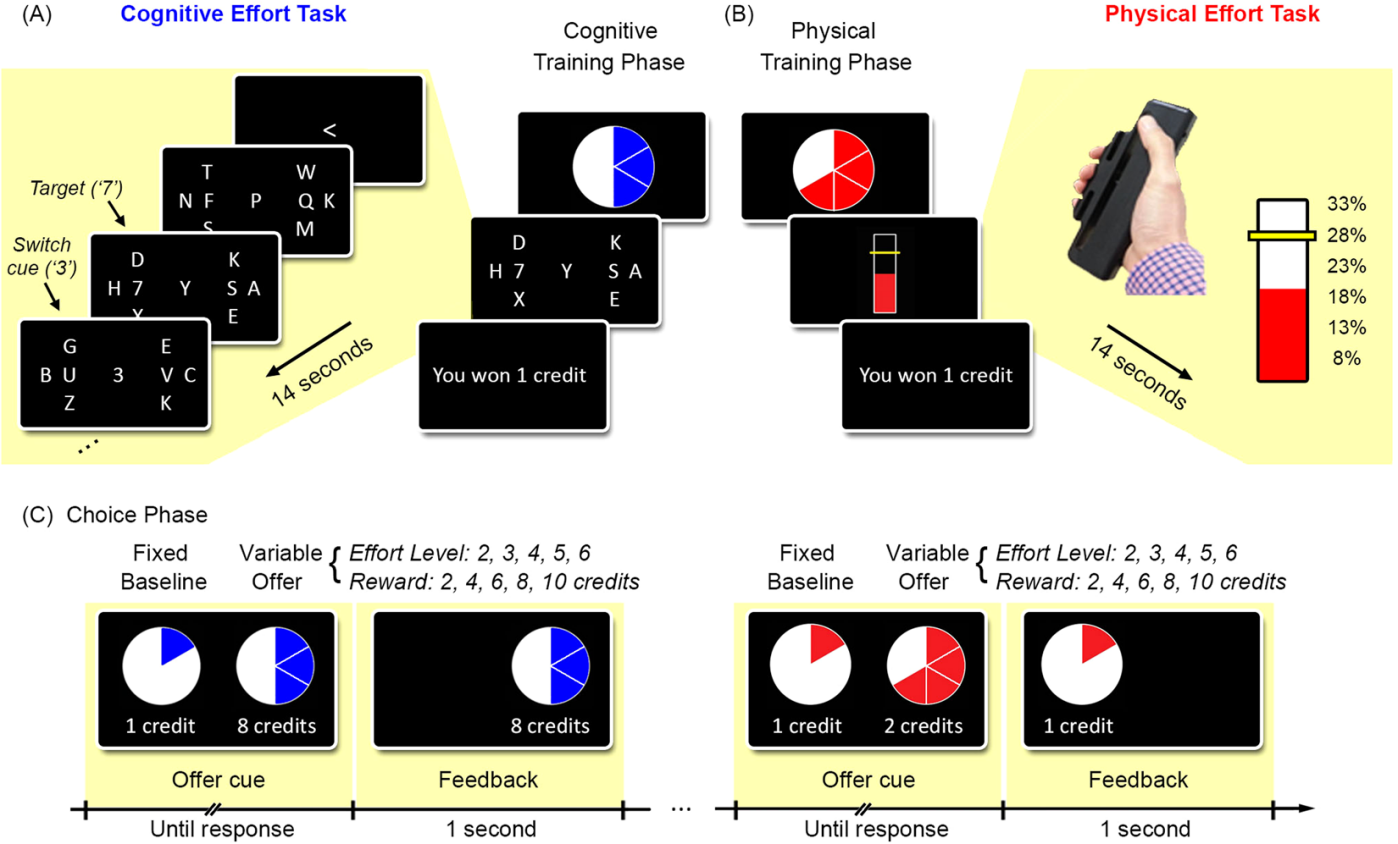
Experimental paradigm. Participants were trained on two separate tasks which parametrically manipulated (**A**) cognitive effort, and (**B**) physical effort. Each trial began with a blue or red pie-chart indicating the up-coming level of cognitive or physical effort, respectively. (**A**) The cognitive effort task required participants to detect a ‘7’ in one of two target RSVP streams to the left or right of fixation (denoted by ‘F’ and ‘Q’ in this example). The target streams were each surrounded by three task-irrelevant distractor streams. An arrowhead at the beginning of the trial indicated the initial target stream. While monitoring the target stream, participants also had to monitor the central stream for a cue (‘3’) to switch their attention to the alternate target stream. We manipulated cognitive effort as the number of times individuals had to switch their attention from one target stream to the other. (**B**) In the physical effort task, individuals were required to maintain a sustained force on a hand-held dynamometer at one of six levels of force, indexed to their specific MVC. (**C**) In the choice phase, participants indicated their preference between a fixed, low-effort/low-reward baseline, and a variable high-effort/high-reward offer.

The experimental paradigm has been described previously^8^. Participants were first trained on two separate tasks – one in which we parametrically varied cognitive effort while holding physical demands constant, and the other in which we varied physical effort while holding cognitive demands constant (Fig. 1A,B). After training, participants undertook the critical choice phase, during which they made economic decisions based on the amount of effort they were willing to trade off in return for varying amounts of reward (Fig. 1C).

The *cognitive effort* task utilised a rapid-serial-visual presentation (RSVP) design. Participants had to fixate on a central letter stream, while monitoring one of two other RSVP streams on either side for a target ‘7’ (Fig. 1A). Three task-irrelevant distractor streams surrounded each of the two target streams^35^. At the beginning of the trial, a central arrow cued participants to the initial target stream (left/right). During the trial, participants had to continue monitoring the central stream for further cues (the number ‘3’) to switch their attention to the opposite target stream. We parametrically varied cognitive effort by increasing the number of times (1 to 6) that attention had to be switched between the left/right target streams.

In the *physical effort task*, participants had to exert one of six levels of force on a hand-held dynamometer (Fig. 1B). To standardise force requirements across individuals, effort levels were defined as percentages of each participant’s maximum voluntary contraction (MVC; 8, 13, 18, 23, 28, 33%). MVCs were determined for each participant at the beginning of the study. Note that cognitive and physical effort trials were identical in duration (14 seconds) to eliminate the effect of temporal discounting on later choice behaviour^36,37^.

The order of cognitive and physical effort tasks was counterbalanced across participants. All participants completed 60 trials (10 per effort level) in each task to reinforce behaviour. Participants were awarded one credit for each successfully completed trial, and were informed that these credits would contribute to their payment at the end of the study. The amount of training ensured that participants were rewarded on almost every trial at each level of effort.

Following the training phase, participants engaged in the critical choice phase (Fig. 1C). On each trial, participants were required to choose between a fixed low-effort/low-reward ‘baseline’, and a variable high-effort/high-reward ‘offer’. The baseline was associated with the lowest level of effort for the lowest reward (1 credit), whereas the offer was associated with a greater amount of effort (Levels 2–5) in return for greater reward (2, 4, 6, 8, 10 credits). Decisions were made separately for the cognitive and physical effort tasks. To eliminate the effect of fatigue, participants were only required to reveal their preferences, but were not required to execute them. Instead, they were told that ten of their choices from each domain would be randomly chosen for them to execute at the conclusion of the experiment, and their remuneration would be based on these trials.

#### Behavioural Training

**Figure 2:**
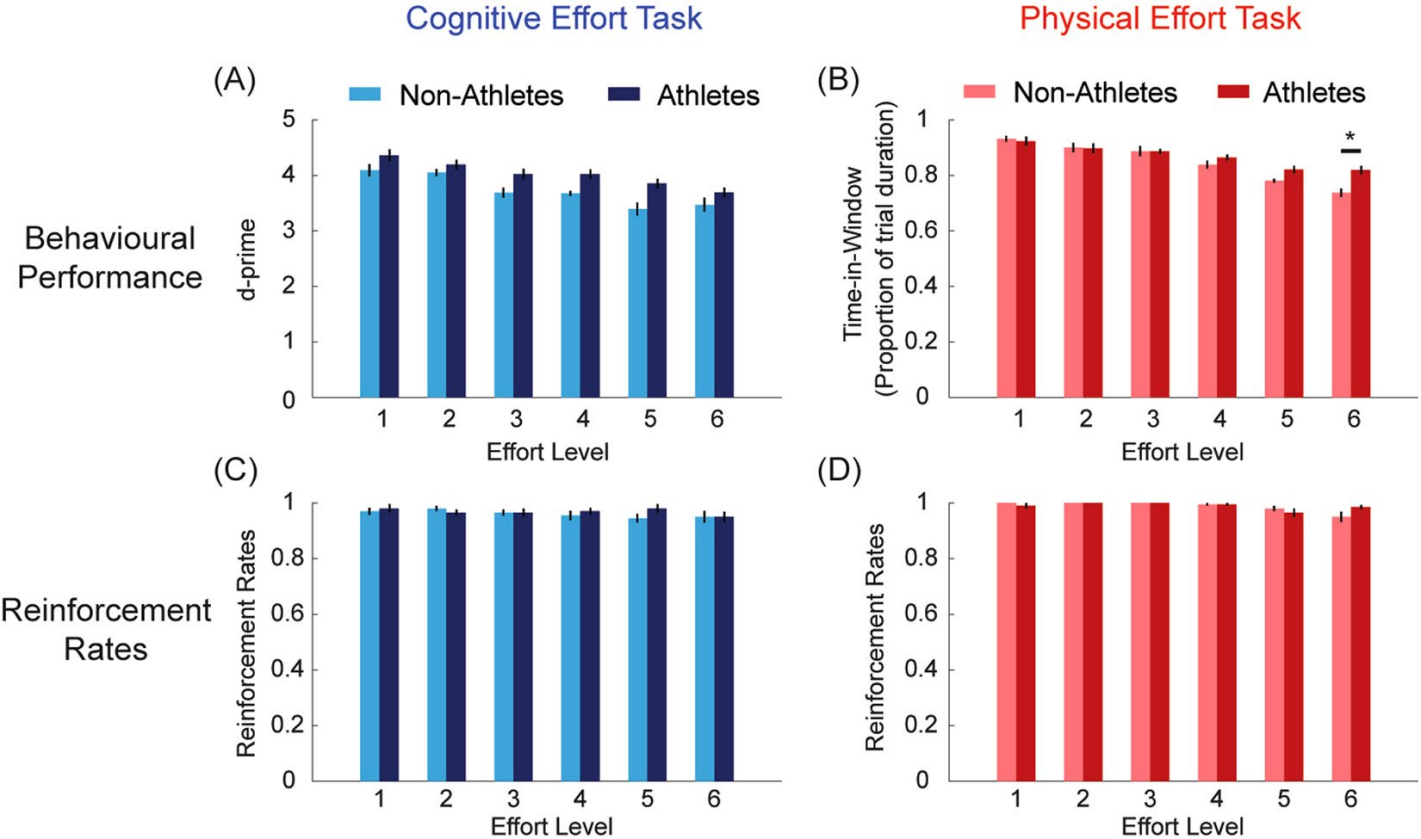
Results from the cognitive (blue) and physical (red) training phase. Athletes are indicated in darker colours, and non-athletes in lighter colours. (**A**,**B**) Objective performance (*d*′ in the cognitive effort task, and % time-in-window in the physical effort task) decreased in both the (**A**) cognitive and (**B**) physical tasks as a function of effort. This confirmed the ability of our paradigms to modulate task demand in the corresponding domains. The only group difference was at the highest level of physical effort. (**C**,**D**) Despite increasing cognitive/physical load, there were no group differences in reinforcement rates in either the cognitive (**C**) or physical (**D**) tasks.

First, we confirmed that our cognitive and physical effort manipulations were successful in objectively increasing task load (Fig. 2). In the cognitive effort task, we operationalised performance in terms of target detection sensitivity, *d*′ (*Z*(Hit) − *Z*(False alarm)) (Fig. 2A). We compared *d*′ as a function of Group (non-athletes, athletes) and Effort Level (1–6) in a mixed-design repeated measures ANOVA. This demonstrated a significant main effect of Effort (*F*(5, 190) = 22.2, *p* < 0.001), with Bonferroni-corrected pairwise comparisons indicating that *d*′ progressively reduced with increasing effort. Importantly, however, neither the effect of Group, nor the two-way interaction, was significant (Group, *F*(1, 38) = 3.03, *p* = 0.09; interaction, *F*(5, 190) = 1.00, *p* = 0.42).

In the physical task, performance was measured as the proportion of time in each trial that participants were able to maintain their force within the target window (Fig. 2B). The analogous ANOVA revealed a significant main effect of Effort, and a Group × Effort interaction (Effort, *F*(5,190) = 92.1, *p* < 0.001; Effort × Group, *F*(5,190) = 8.09, *p* < 0.001). This showed an overall decrease in performance with increasing effort across both groups, with a group difference only at the highest effort level, at which athletes were able to maintain their force for longer than non-athletes (athletes 82 ± 1.8%, vs non-athletes 73.8 ± 1.8%, *p* = 0.002). Together, these analyses confirm that our cognitive and physical effort paradigms were effective in modulating task load.

Finally, we wished to confirm that, despite the decrements in performance with increasing effort, there were no group differences in the ability to be successfully rewarded at each effort level (Fig. 2C,D). Reinforcement rates were calculated as the proportion of trials that individuals were successfully rewarded at each effort level. Overall reinforcement rates for both tasks were very high (96.5% for cognitive effort; 98.8% for physical effort). In the cognitive effort task, a Group × Effort ANOVA did not reveal any significant results (Effort, *F*(3.8, 145) = 1.04, *p* = 0.39; Group, *F*(1,38) = 0.29, *p* = 0.59; Effort × Group *F*(3.8, 145) = 0.96, *p* = 0.43). The analogous ANOVA on the physical effort task showed a significant main effect of Effort, indicating differences between low and high levels (*F*(2.6, 99.4) = 6.6, *p* = 0.001; level 2 > 6 and level 3 > 5, both *p* = 0.04)). Importantly, however, neither the effect of Group, nor its interaction with Effort, were significant (Group, *F*(1,38) = 0.07, *p* = 0.79; Group × Effort, *F*(2.6, 99.4) = 2.4, *p* = 0.08).

Overall, these analyses indicate that: (i) both the cognitive and physical effort tasks were successful in manipulating load, and (ii) despite the increase in load, there were no group differences in the rate at which individuals were reinforced at each level of effort.

#### Choice Behaviour – Effort and Reward Sensitivity

**Figure 3:**
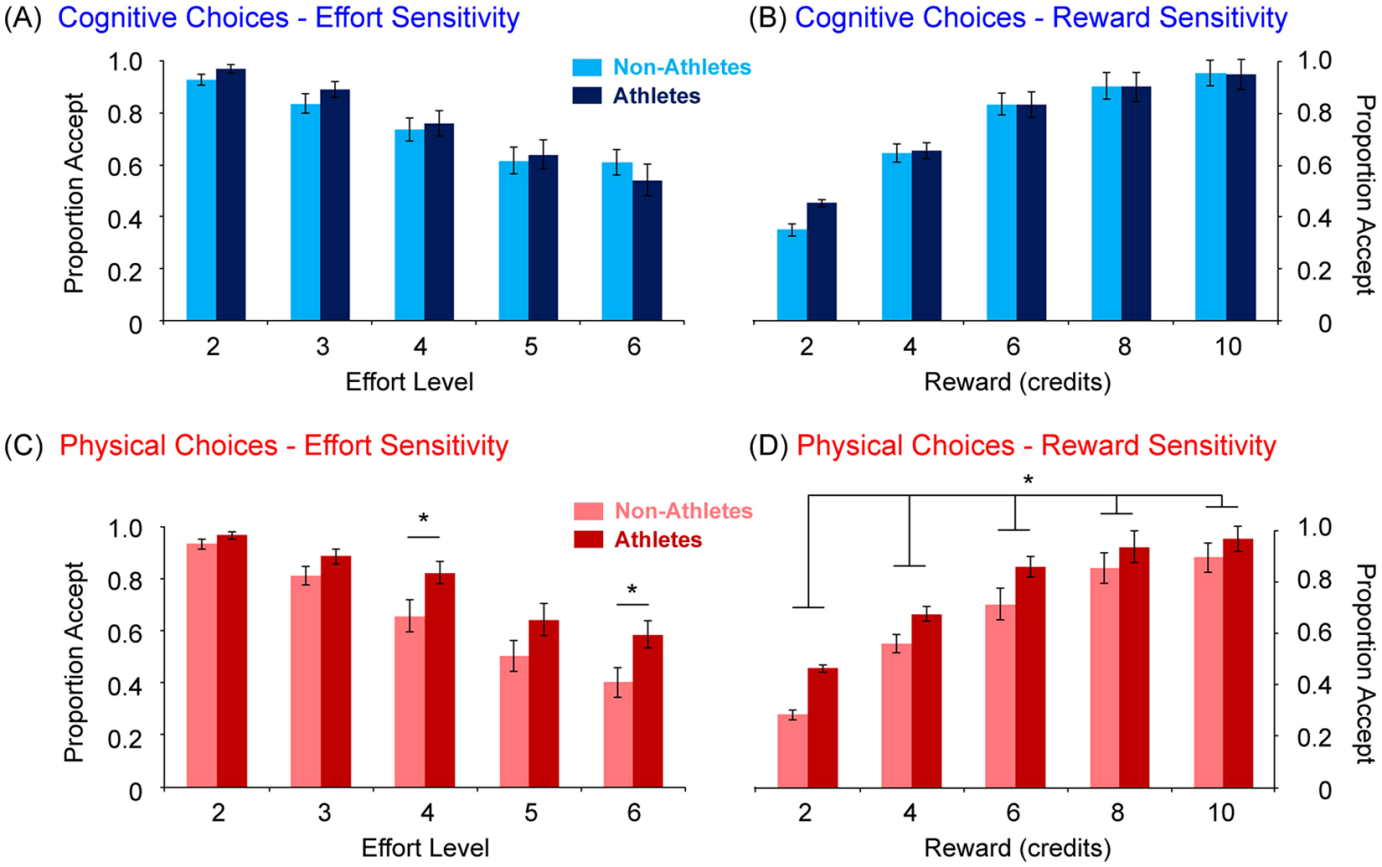
Behavioural data revealed differences between athletes and non-athletes in physical, but not cognitive, effort discounting. Proportion of accepted offers are shown as a function of (**A**,**C**) Effort and (**B**,**D**) Reward for the (**A**,**B**) Cognitive Effort and (**C**,**D**) Physical Effort tasks. Athletes chose the higher effort levels more frequently than non-athletes (Levels 4 and 6), for which they needed to incentivised with proportionally greater rewards (*p < 0.05).

Effort Sensitivity. Next, we asked whether there were any behavioural differences in effort sensitivity across the two domains. We analysed the proportion of trials in which participants accepted the more effortful offer, as a function of increasing Effort (Fig. 3A,C). A three-way mixed repeated-measures ANOVA on the between-subjects factor of Group (rowers, non-rowers), and the within-subjects factors of Domain (cognitive, physical) and Effort (2–6) was performed. This showed a main effect of Effort, with higher effort levels chosen consistently less frequently than lower effort levels (*F*(2.39, 76.9) = 120, *p* < 0.001; *p*-values for all pairwise comparisons <0.05).

Although the main effect of Group was not significant (*F*(1,38) = 1.81, *p* = 0.19), it was involved in significant higher order interactions (Domain × Group, *F*(1, 38) = 6.94, *p* = 0.01; Domain × Effort × Group, *F*(1.90, 72.2) = 5.16, *p* = 0.01). Decomposing these interactions with post-hoc Bonferroni-corrected *t*-tests revealed that athletes and non-athletes differed only in effort sensitivity for the higher levels of physical effort (level 3, *p* = 0.03; level 5, *p* = 0.02), but there were no significant group differences in choice for any level of cognitive effort (all *p*-values > 0.14). The remaining main effects and interactions were not significant (Domain, *F*(1,38) = 2.46, *p* = 0.125; Effort × Group, *F*(2.02, 76.9) = 0.51, *p* = 0.60; Domain × Effort, *F*(1.90, 72.2) = 2.27, *p* = 0.11). Together, this suggests a difference in effort sensitivity between athletes and non-athletes only at the higher levels of physical effort.

Reward Sensitivity. The complementary analysis on offer acceptance as a function of Reward revealed a significant main effect of Reward (*F*(2.29,87.2) = 136.7, *p* < 0.001), such that successive levels of reward were chosen increasingly frequently (all *p* < 0.05; Fig. 3B,D). There was also a significant main Domain × Group interaction (*F*(1,38) = 6.05, *p* < 0.05), which indicated that athletes were more inclined to accept all levels of reward than non-athletes, but only when the effort required was physical (*p* = 0.03), and not cognitive (*p* = 0.71). No other main effects or interactions were significant (Domain, *F*(1,38) = 1.95, *p* = 0.17; Reward × Group (*F*(2.30, 87.2) = 1.22, *p* = 0.31; Domain × Reward, *F*(2.77,105.4) = 0.63, *p* = 0.65; three-way, *F*(2.77, 105.4) = 0.70, *p* = 0.54). Together, these results indicate that the two groups differed in choice preference only in the physical, and not the cognitive, domain.

#### Logistic Regression of Choice Behaviour

Next, we wished to verify that participants’ choices were not merely driven by risk aversion (i.e., a lower likelihood of accomplishing the higher levels of effort). This was unlikely to be the case, given the very high reinforcement rates (>95%) in the training phase of both tasks. Nevertheless, to definitively confirm that the probability of succeeding at each level did not affect choice preference, we performed a logistic regression on the effect of reinforcement rates, rewards and effort on choice (Supplementary Data). Importantly, this analysis revealed that reinforcement rates did not predict choice behaviour in either domain (cognitive, *p* = 0.28; physical, *p* = 0.53), but that effort and reward did so in the predicted directions in both domains (all *p*-values < 0.001). We also asked whether performance itself (i.e., *d*′ for the cognitive task, and time-in-window for the physical task), in addition to effort and reward, could account for choices. This analysis again revealed that performance did not affect choice (cognitive, *p* = 0.41; physical, *p* = 0.99). Together, these analyses confirm that the effort discounting seen in our study was driven by an aversion to effort, rather than probability discounting.

#### Computational Modelling of Choice Behaviour

The behavioural data showed an expected difference in physical motivation between athletes and non-athletes, with little difference in cognitive motivation. However, our previous work using the same paradigm has shown that computational models may be a more sensitive approach to probing differences in choice behaviour^8^. Thus, using a similar approach, we asked whether the *pattern* of effort discounting differed across groups. Although many functions have previously been used to computationally model effort discounting^15,16,18,38,39^, three functions were selected to capture the patterns that we predicted based on our recent study with the identical paradigm^8^. Specifically, we used linear, hyperbolic and parabolic functions to fit choices made in both tasks:

1 $${Linear}:SV(t)=R(t)\cdot (1-k\cdot E(t))$$

2 $${Hyperbolic}:SV(t)=R\,(t)\cdot \frac{1}{1+k\cdot E(t)}$$

3 $${Parabolic}:SV(t)=R(t)-k\cdot E{(t)}^{2}$$

where S*V*(*t*) is the subjective value of the offer on trial *t*; *R* is the reward in credits (2, 4, 6, 8, 10); *E* is the effort level (0.2, 0.4, 0.6, 0.8, 1.0); and *k* is a subject-specific effort discounting parameter, with higher *k* values indicating steeper discounting functions. Each participant’s discounting function is referenced to the SV of the baseline offer (one).

The different shapes of these three functions reflect how increasing effort affects choice behaviour. Linear models imply constant discounting as effort increases; hyperbolic (convex) models predict that changes at lower levels of effort will have greater impact than changes at higher levels; and parabolic (concave) models predict the opposite. These three functions capture the main patterns of effort discounting, with the hyperbolic and parabolic specifically motivated by our recent study which found them to describe cognitive and physical effort discounting, respectively^8^. Note that each of these functions contained the identical number of free parameters, allowing subsequent model comparisons to be interpreted unambiguously.

For each group, we fitted these functions to choices in each of the two domains (i.e., 3^2^ = 9 models each for the athlete and non-athlete groups). Within each group, we then compared these nine different models to examine whether cognitive and physical effort costs have a differential effect on reward devaluation. Models were fit using a *softmax* function and maximum likelihood estimation, with the *softmax* function being defined as:

4 $${\rm{\Pr }}(i)=\,\frac{{e}^{\beta \cdot S{V}_{i}}}{{e}^{\beta }+{e}^{\beta \cdot S{V}_{i}}}$$

where Pr(*i*) represents the probability of choosing option *i*, *SV*_*i*_ is the subjective value of *i*, and *β* is the inverse temperature defining choice stochasticity. For each model, the effort discounting parameter (*k*) and the inverse temperature of the *softmax* function (*β*) were modelled separately for each domain^8^.

**Figure 4:**
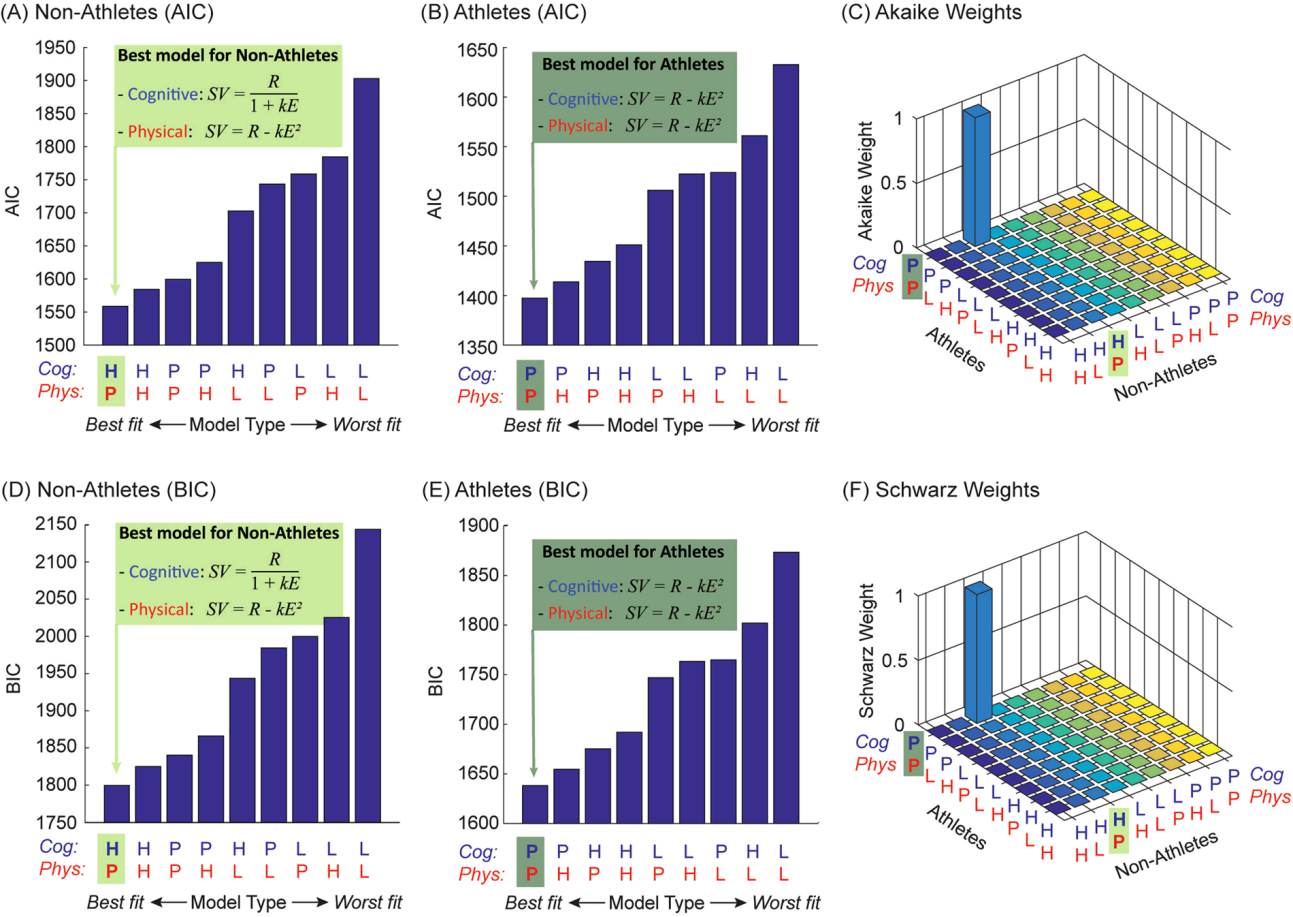
Model comparisons with an AIC (**A**–**C**) and BIC (**D**–**F**). Data are plotted separately for non-athletes (**A**,**D**) and athletes (**B**,**E**). We compared models that assumed: a hyperbolic (H) linear (L) or parabolic (P) effort discounting function for the cognitive and physical tasks. Results are ordered according to increasing AIC or BIC, with lower values indicating better model fits. The winning model for non-athletes described cognitive effort discounting as a hyperbolic function and physical effort discounting as a parabolic function. In contrast, the winning model for athletes described both cognitive and physical effort discounting as parabolic functions. (**C**) This combination of results (hyperbolic/parabolic fits for non-athletes; parabolic/parabolic fits for athletes) had Akaike and Schwarz weights in excess of 0.99, indicating high likelihoods of it being the best fitting model.

We compared model fits for each group with an Akaike Information Criterion (AIC) and a Bayesian Information Criterion (BIC) (see Supplementary Figs 2–5 for individual model fits)^8^. Overall, this analysis showed different patterns of effort discounting in athletes and non-athletes (Fig. 4). In particular, the best fitting model for non-athletes showed that cognitive effort discounting followed a hyperbolic pattern, and physical effort discounting a parabolic pattern (Fig. 4A,D). Note that this is identical to the winning model in the original paper that described this task on an unselected population^8^. Interestingly, however, this model comparison revealed a different pattern of effort discounting in athletes. Specifically, a parabolic function best fit the pattern of effort discounting, not only in the physical domain (as in non-athletes), but also in the cognitive domain (Fig. 4B,E).

To quantify the likelihood that these separate models for athletes and non-athletes best accounted for choice behaviour across the entire group, we computed the Akaike weights for each of the 9^2^ = 81 models across the entire model space (Fig. 4C). Akaike weights represent the relative likelihood of a model relative to other models in the space, and is given by:

5 $${w}_{i}(AIC)=\,\frac{{e}^{-0.5\cdot {{\rm{\Delta }}}_{i}(AIC)}}{{\sum }_{m=1}^{M}{e}^{-0.5\cdot {{\rm{\Delta }}}_{m}(AIC)}}$$

where w_i_(AIC) = the Akaike weight of model *i*; Δ_i_(AIC) = the difference in AIC between model *i* and the best fitting model; and *M* = the number of models in the space. This analysis revealed that the relative likelihood that this combination of model fits best explained motivation across the group was 0.9997. The analogous computation for BICs was undertaken with Schwarz weights, which yielded the equivalent result (Fig. 4F).

To confirm that the group differences in *k* value for physical effort reflected the behavioural finding of greater willingness to invest effort in athletes vs non-athletes, we conducted a two-sample *t*-test between the two groups. This showed significantly greater *k*-values (and therefore lower physical motivation) for non-athletes relative to athletes (non-athletes, 7.68 ± 1.21, vs athletes, 4.77 ± 0.73; *t*(38) = 2.1, *p* = 0.046; Supplementary Fig. 6). This confirms the basic behavioural finding that showed a greater willingness to exert physical effort in rowers vs non-rowers. Note that no direct comparison can be made of the cognitive effort discounting parameters between groups because of the different functions involved. For completion, we also compared the inverse temperatures (β) between groups for each domain, but this did not yield any significant group differences (cognitive task: non-athletes, 17.7 ± 6.53, vs athletes, 8.19 ± 4.18; *t*(38) = 1.17; physical task: non-athletes 1.20 ± 0.15, vs athletes, 12.1 ± 5.71, *t*(38) = 1.91).

Overall, these analyses lead to two conclusions. First, rowers and non-rowers follow similar discounting patterns for physical effort, but the former are more motivated to invest effort than the latter. Second, there is a fundamental difference in the way in which cognitive effort is discounted between the two group (concave for athletes and convex for non-athletes). This suggests a complex relationship between cognitive and physical motivation, such that greater physical motivation is not merely associated with an increase in cognitive motivation, but a fundamental difference in the pattern of effort discounting in the cognitive domain.

#### Subjective Perception of Mental and Physical Demand

**Figure 5:**
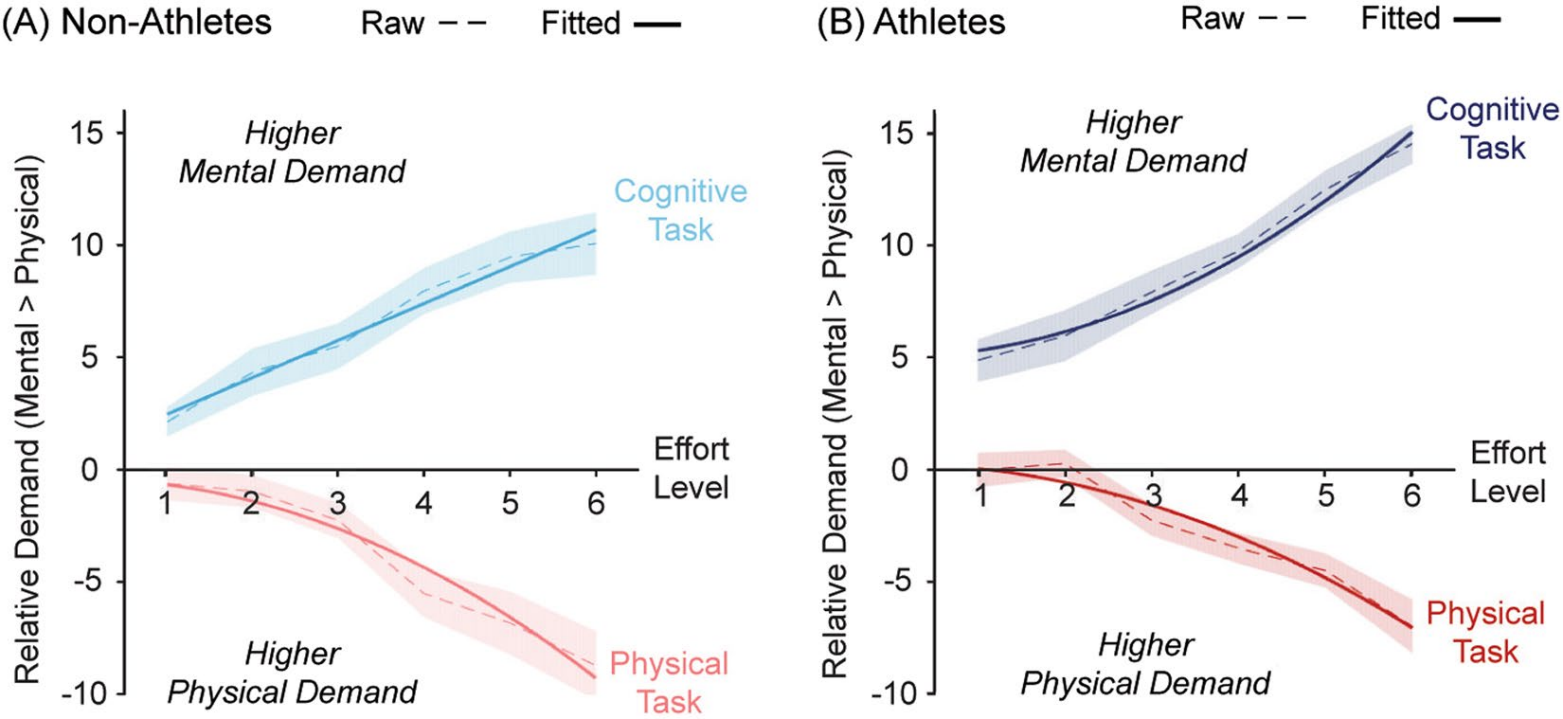
The subjective perceived demand of the cognitive and physical effort tasks increased in the corresponding domain for both the (**A**) Non-athletes, and (**B**) Athletes. The ordinate is a difference score calculated as Mental > Physical demand for each level of effort on the NASA Task Load Index. Positive values therefore indicate greater perceived mental relative to physical demand. All demand curves were best fit by parabolic functions, except for the relative mental demand of the cognitive task by non-athletes, which was best fit by a linear function. This pattern of results recapitulates the findings for subjective value.

Was the difference in cognitive effort discounting between the two groups accompanied by differences in perceived cognitive demand? At the conclusion of the experiment, we administered the NASA Task Load Index to ascertain participants’ perception of subjective task demands. Specifically, we asked them to provide ratings of the perceived ‘mental demand’ and ‘physical demand’ of each of the six effort levels, in each of the two effort tasks, on a 21-point scale (Supplementary Data). For each task, we subtracted scores on the Physical from the Mental Demand subscales, in order to derive a single metric indicating the relative mental vs physical demand of each effort level (positive = more mentally-demanding) (Fig. 5). As expected, these data showed that our manipulations of cognitive and physical effort resulted in increases in perceived demand within the corresponding domain, and decreases in demand in the alternate domain.

To determine whether the perceived demand curves differ between groups, we fit non-linear regression models to participants’ effort ratings using linear, parabolic and hyperbolic functions (Supplementary Data). Interestingly, this analysis resulted in similar conclusions to the computational analyses on effort discounting. Specifically, in athletes, parabolic functions described the increases in both the relative mental demand for the cognitive task, and relative physical demand for the physical task (Fig. 5B). Importantly, however, the data from non-athletes showed a parabolic increase in the relative physical demand of the physical task, but a different function (in this case, linear) described increases in relative mental demand in the cognitive task (Fig. 5A). Together, this pattern of results recapitulates the computational findings, and provides independent evidence to support a difference in effort-based decision-making between athletes and non-athletes.

## Discussion

To our knowledge, this is the first study to examine effort discounting in elite athletes. Although an extensive literature in sport psychology has emphasised the importance of motivational factors in the athletic pursuit of reward^40–42^, little is known about the mechanisms that underlie the greater motivation of athletes relative to non-athletes, either within or across separate domains of effort. Here, our computational models of motivated behaviour revealed three key findings. First, as predicted, athletes were more physically motivated than their non-athletic counterparts. Second, this increase in physical motivation was accompanied by a fundamentally different pattern of cognitive motivation between the two groups. Finally, the altered pattern of cognitive motivation in athletes relative to non-athletes was accompanied by corresponding changes in the perceived demands of the cognitive task. Together, these results show that a higher degree of motivation in one domain (physical) can be associated with altered motivation in a separate domain (cognitive).

The results of the physical effort task are consistent with previous reports. Several studies have shown that physical effort discounting is well-described by parabolic functions, although these earlier studies did not systematically examine the effect of individuals’ baseline levels of motivation^8,15,43,44^. Here, we found that physical effort discounting in both athletes and non-athletes was described by the same concave function, which differed only in its slope. One obvious reason for the shallower effort discounting in athletes is that they simply found the physical effort task to be less demanding than non-athletes. Indeed, there was some suggestion of this in the training data, which showed that, at the highest level of effort, athletes were able to maintain their force within the required window for longer than non-athletes. Interestingly, however, the subjective physical demand ratings for the physical effort task did not significantly differ between groups (Fig. 5 and Supplementary Fig. 7). This finding that athletes are more willing to invest physical effort than non-athletes, despite similar levels of perceived physical demand, is consistent with several motivational theories of sporting performance, which suggest that athletic success is characterised by high degrees of intrinsic motivation^45^, greater perceived competence^46^, and/or higher self-efficacy^47,48^.

In contrast, cognitive effort discounting was distinctly different between groups. In non-athletes, the hyperbolic effort discounting function replicated previously published findings using the identical task in a sample of unselected participants^8^. The convexity of this function suggests an intolerance in controls of even low levels of cognitive load, with steeper reward devaluation occurring at the lower levels of cognitive effort. However, the opposite was true of athletes. Their concave discounting pattern implied that they were more tolerant of low levels of cognitive load, with rewards mainly being devalued at higher levels of effort^16^ – a pattern that mirrored the parabolic increase in their perception of relative mental demand (Fig. 5B, blue line). It is worth noting that this greater tolerance of low effort by athletes generalised across both domains, even extending to a (cognitive) task in which they had not been specifically trained. This builds on applied research in sport psychology, which typically focuses on the capacity of athletes to exert effort in a form more closely related to their particular field (e.g., examining physical capacity in cyclists with a cycling task)^24^. From a neuroeconomic perspective, our results suggest that the way in which cognitive effort costs are integrated in making a decision may be quite unlike other forms of economic discounting, which are more consistently described by a single function (e.g., hyperbolic functions for delay discounting^36,37^). Instead, changes in cognitive motivation may be manifest as, not merely changes in the effort discounting gradient, but also in the shape of the function itself.

It is important to note that differences in effort discounting between the two groups cannot be due to confounding factors such as temporal or probability discounting. First, the temporal parameters of the cognitive and physical effort tasks were identical^16,49,50^. In addition, group differences are unlikely to have been driven by a differential ability in being successfully rewarded. We note that there were possible small group differences in performance in the training phase. For example, during physical effort training, athletes were able to maintain their force for slightly longer than non-athletes, but only at the highest effort level; and there was also a trend towards higher *d’*s in athletes vs non-athletes in the cognitive task (*p* = 0.09). Importantly, however, the probability of each group being successfully rewarded at each effort level (i.e., the reinforcement rates) was at ceiling, and similar across all levels of effort for both groups (notwithstanding a trend towards a Group x Physical Effort interaction, *p* = 0.08). A further critical point to note is that logistic regressions showed that neither reinforcement rates nor behavioural performance significantly influenced choice behaviour. Overall, therefore, our results isolate the effect of subjective value on motivation from many effects that can confound studies examining effort-based decisions.

Together, these results provide a mechanistic basis for recently postulated psychobiological models of sporting performance^25–27^. These models are based on motivational intensity theory^51^, with a core feature being that sporting performance is based on the willingness of an athlete to exert effort to achieve a reward. By modelling participants’ decisions as a function of subjective value, our approach provides a computational account of how such effort-based decisions might be instantiated. Complementing these data are the results of a recent fMRI study, which used the identical paradigm in a group of unselected adults^8^. This fMRI study suggests that the effort-based decisions made in the present study are likely subserved by a network of domain-general areas (including the dorsomedial prefrontal cortex and dorsal anterior cingulate cortex), as well an area that uniquely encoded cognitive effort-based choices (the amygdala).

An outstanding question is how each of these nodes are selectively influenced by targeted training in one domain over another. An obvious interpretation for our data is that physical training not only augmented physical motivation in athletes, but also carried over to influence other domains of effort. An alternative, non-mutually exclusive, explanation is that, relative to those who choose not to pursue an athletic career, those who become elite athletes are inherently more driven. Future interventional studies could adjudicate whether the greater motivation in athletes relative to non-athletes seen here is cause or consequence of their intensive physical training, by prospectively applying paradigms such as ours following targeted interventions in the cognitive and/or physical domains.

In summary, this study reveals a complex relationship between cognitive and physical motivation, in which higher motivation in one domain may be associated with altered patterns of motivation in the alternate domain. More broadly, these results have clinical implications for conceptualisations of disorders of motivation, such as apathy, in patient populations^52,53^. Currently, apathy is considered to consist of different subtypes (e.g., behavioural, cognitive and emotional)^54–58^. A question that remains for future studies is to examine how interventions aimed at improving motivation in one domain may affect motivation in the other – a possibility which could have important implications for rehabilitative programs involving cognitive and physical training in patients, as well as the elderly.

## Methods

### Cognitive Effort Task

The cognitive effort task was based on a previously described RSVP paradigm^35^, and implemented in Presentation (www.neurobs.com). Participants had to fixate on a central RSVP stream while simultaneously monitoring two other RSVP streams to the left and right of fixation for a target ‘7.’ The target streams were each surrounded by three task-irrelevant distractor streams. Trials began with a blue pie-chart, which cued the level of effort associated with that trial. The initial target stream was designated by a left or right arrowhead at fixation. While monitoring that target stream, participants had to simultaneously monitor the central stream for a ‘3’, which was the cue to switch their attention to the opposite target stream. We operationalised cognitive effort as the number of times that participants had to reallocate their attention on each trial, which could vary from one to six times^5,8^.

Participants performed 18 practice trials (three at each of the six levels of effort), followed by 60 trials in the training phase of the experiment (10 at each effort level). Each trial comprised 40 serially presented letter stimuli, each of which lasted 350 ms, for a total trial duration of 14 seconds. The central switch cue (‘3’) occurred at pseudo-random intervals. There were three targets per trial, and participants indicated their response by button press. Participants were required to detect at least one of the three targets, and commit fewer than two false alarms, to be rewarded one credit. Feedback was given at the end of each trial, and participants were informed that these credits would be used to determine their remuneration.

Note that the advantages of this paradigm are: (1) it is strongly motivated by a large literature establishing the relationship between attentional load and cognitive effort^35,59^, and is well-established in studies of attentional load^35^; (2) it allowed us to parametrically vary effort over multiple levels; and (3) its efficacy in eliciting cognitive effort discounting has been established in recently published studies^5,8^.

### Physical Effort Task

In the physical effort task, participants squeezed a hand-held dynamometer at one of six different levels of force with their dominant (right) hand. The dynamometers (SS25LA, BIOPAC systems, USA) were interfaced with a computer running Psychtoolbox (http://psychtoolbox.org) as implemented in Matlab (Mathworks, USA). Prior to the task, each participant’s maximum voluntary contraction (MVC) was determined as the maximum contraction reached over three trials.

The task required participants to maintain a constant grip force at one of six levels of effort, which were standardised across participants as percentages of their MVC (8%, 13%, 18%, 23%, 28%, 33%). Trials began with a red pie-chart indicating the force required on that trial. A vertical bar then appeared on the screen, with a yellow horizontal line indicating the target force level. During the trial, participants received visual feedback on their exerted force. For participants to be rewarded, they were required to maintain their force within 2.5% of the target force for ≥50% of the 14-second trial duration. Note that the trial durations in both the cognitive and physical effort tasks were identical.

As in the cognitive effort task, subjects first undertook 18 practice trials (three per effort level). They then completed the training phase, which comprised 60 trials (10 trials at each effort level). Each successfully completed trial earned participants one credit, and participants were provided with feedback at the end of each trial. The order of cognitive and physical training blocks was counterbalanced across participants.

### Choice Period

After the training phase, participants entered the critical decision-making period. On each trial, participants were provided with two options, and asked to choose whichever was preferable to them. One option was a ‘baseline’ low-effort/low-reward option (Effort Level 1 for 1 credit). This fixed baseline was always contrasted against a high-effort/high-reward ‘offer’, which comprised a higher level of effort (Effort Levels 2–6) for a higher reward (2, 4, 6, 8, 10 credits). The baseline and offer were always within the same domain. Participants were told that ten choices from each domain would be randomly sampled for them to perform at the conclusion of the experiment, and that this would determine their remuneration. All participants were paid £30, but a debriefing following the study showed that participants all believed that their payment depended on their choices.

The order of cognitive and physical effort choices was randomised. The entire effort-reward space was sampled randomly and evenly, with all points in that space sampled three times per domain. Trials were self-paced, and each offer remained on the screen until participants registered their responses. The ‘baseline’ option was positioned on the left side of the screen, and the ‘offer’ on the right. Participants registered their response with a left or right button press, upon which their selected response would be highlighted on the screen for one second.

### Data availability

The datasets generated during and/or analysed during the current study are available from the corresponding author on reasonable request.

## Electronic supplementary material

Supplementary Information
