# Supplementary material for: Computational modelling reveals distinct patterns of cognitive and physical motivation in elite athletes
Source: Sci Rep. 2018 Aug 8;8:11888. doi: 10.1038/s41598-018-30220-3 (PMC6082862; doi:10.1038/s41598-018-30220-3)
Supplement: Supplementary file 1 — Supplementary Information [file 41598_2018_30220_MOESM1_ESM.docx]

**Computational modelling reveals distinct patterns of cognitive and physical motivation in elite athletes**

**Supplementary Data**

Trevor T-J Chong ^a^, Matthew A J Apps ^b^, Kathrin Giehl ^d^, Stephanie Hall ^b^, Callum H Clifton ^b^, & Masud Husain ^b,c^

^a^ Monash Institute of Cognitive and Clinical Neurosciences, Monash University, Victoria 3800, Australia

^b^ Department of Experimental Psychology, University of Oxford, Oxford OX1 3UD, United Kingdom

^c^ Nuffield Department of Clinical Neurosciences, John Radcliffe Hospital, Oxford OX3 9DU, United Kingdom

^d^ Department of Nuclear Medicine, University of Cologne, 50937 Cologne, Germany

**Choice Behaviour**

An alternate approach to analyse choice behaviour, which incorporates both effort and reward sensitivity, is to calculate the proportion of trials that participants chose the high-effort/high-reward offer relative to baseline, and then compute the **area-under-the-curve (AUC)** of this 5 x 5 (effort x reward) space. An ANOVA on the factors of Group and Domain revealed an interaction (*F*(1,38) = 6.53, *p* = .02), such that the AUC for the physical effort task was greater for athletes relative to non-athletes, indicating an overall higher amount of motivation (athletes, 19.5 ± 0.90, vs non-athletes, 16.9 ± 0.90, *p* < .05). However, there were no group differences in the cognitive effort task (athletes, 19.0 ± 0.90, vs non-athletes, 18.9 ± 0.90, *p* = .95). This result is consistent with the analyses reported in the main text, which show significant model-free group differences in the physical effort task, but not the cognitive effort task.

**Logistic Regression of Choice Behaviour**

We sought to ensure that participants’ choices could not be explained by a lower likelihood of succeeding at the higher effort levels (i.e., probability discounting). Overall, this was unlikely, as overall reinforcement rates were >95% for each of the cognitive and physical effort tasks. Nevertheless, we performed a logistic regression on choice data to verify that reinforcement rates in the training phase did not affect participants’ preferences (**Supplementary Figure 1A-B**). We chose a logistic regression because of the binary nature of the key dependent variable (choice) ^1^. For each participant, we entered choice (baseline = 0; offer = 1) as the outcome, with predictor variables of reinforcement rates, effort level, and reward level. This therefore allowed us to examine whether any parameter could explain behaviour significantly over and above any correlations with other variables.

The regression took the form of a standard logistic function, namely:

$p= \frac{1}{1+e^{-logit\left( p \right)}}$

$$logit\left( p \right)=\beta_{0}+\beta_{r}r+\beta_{E}E+\beta_{R}R$$

where *r* (reinforcement rate), *E* (effort) and *R* (reward) are the explanatory variables; $\beta$*_r_*, $\beta$*_E_* and $\beta$*_R_* their respective regression coefficients; and $\beta$*_0_* is a constant. Beta values were then normalised to *t*-statistics as *β*/SE(*β*) to compensate for the possibility of poor *β* estimates in participants with low levels of variance for any variable (although the pattern of results was identical with raw beta values). To determine which of the factors significantly explained choice, we then tested the difference between the *β* value of each covariate against zero. Given that the *β* values were non-normally distributed, we did so with non-parametric Wilcoxon signed-rank tests.

For both the cognitive and physical tasks, Effort level significantly predicted choice behaviour in the negative direction (i.e., higher Effort levels were chosen less frequently; *t_c_*_og_ = -2.24, *p* < .001; *t*_phys_ = -2.48, *p* < .001). Conversely, Reward level significantly predicted choice behaviour in the positive direction (i.e., higher Rewards were chosen more frequently; *t*_cog_ = 2.69, *p* < .001; *t*_phys_ = 2.52, *p* < .001). The most important result, however, was that Reinforcement rates did *not* significantly predict choice (*t*_cog_ = 0.31, *p* = 0.28; *t*_phys_ = 0.14, *p* = 0.53). These analyses indicate that participants’ success at accomplishing each effort level did *not* influence their willingness to engage in those levels. This therefore excludes probability discounting as an explanation for participants’ choices in our study.

**
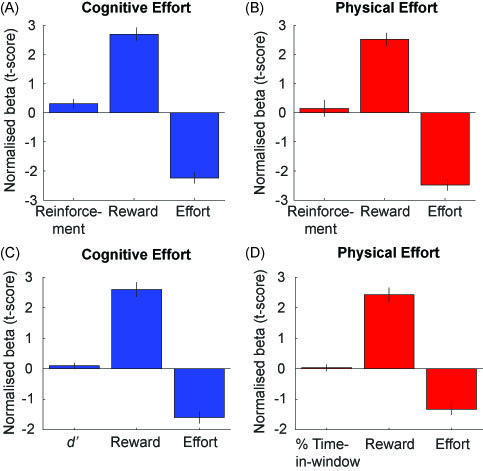
**

**Supplementary Figure 1.** Logistic regressions revealed that reinforcement rates could not account for choice preference in either the (A) cognitive or (B) physical effort tasks. Neither could performance (*d’* in the cognitive task; % time-in-window in the physical task) account for choice preference in either the (C) cognitive or (D) physical effort tasks.

In a separate logistic regression, we examined whether objective task performance (rather than reinforcement rates) could explain choice behaviour (**Supplementary Figure 1C-D**). We quantified performance in the cognitive task as *d’,* and in the physical task as the proportion of time participants maintained their contraction in the required force window. We then used these performance measures, together with effort and reward, as regressors in separate logistic regressions for the cognitive and physical effort tasks.

These analyses yielded the identical pattern of results to those reported above for reinforcement rates. Specifically, for both the cognitive and physical tasks, Effort level significantly predicted choice behaviour in the negative direction (i.e., higher Effort levels were chosen less frequently; *t_c_*_og_ = -1.59, *p* < .0001; *t*_phys_ = -1.33, *p* < .0001). Conversely, Reward level significantly predicted choice behaviour in the positive direction (i.e., higher Rewards were chosen more frequently; *t*_cog_ = 2.60, *p* < .0001; *t*_phys_ = 5.51, *p* < .0001). The most important result, however, was that neither d’ nor time-in-window significantly predict choice (*t_d’_* = 0.10, p = .41; *t*_phys_ = 0.03, *p* = .99).

Together, this indicates that: (1) performance (either as objective performance or as reinforcement rates) does not significantly explain choice behaviour; and (2) effort discounting and reward sensitivity persist even when performance is regressed out.

**Model Fits**

**
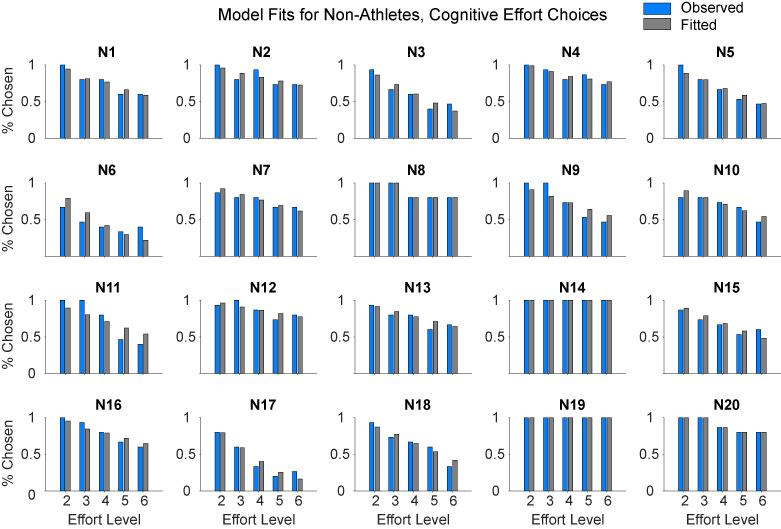
**

**Supplementary Figure 2.** Observed and model-predicted data are plotted for cognitive effort choices for each individual in the non-athlete group. Observed data are depicted in light blue, and fitted data in grey.

**
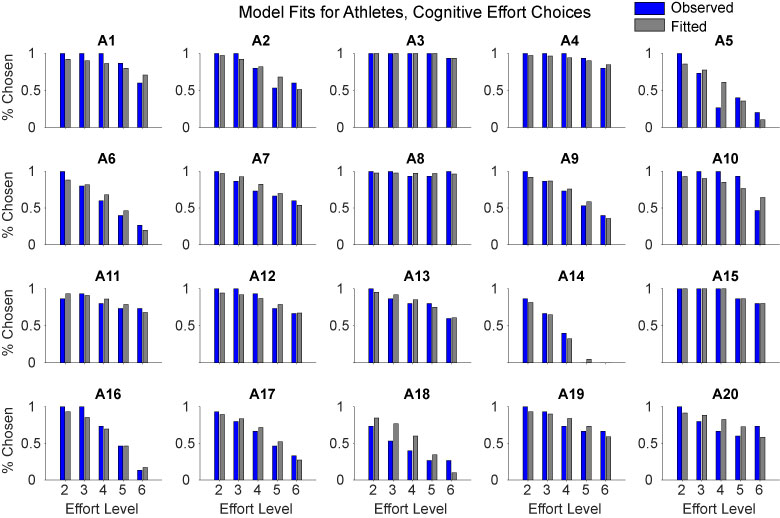
**

**Supplementary Figure 3.** Observed and model-predicted data are plotted for cognitive effort choices for each individual in the athlete group. Observed data are depicted in dark blue, and fitted data in grey.

**
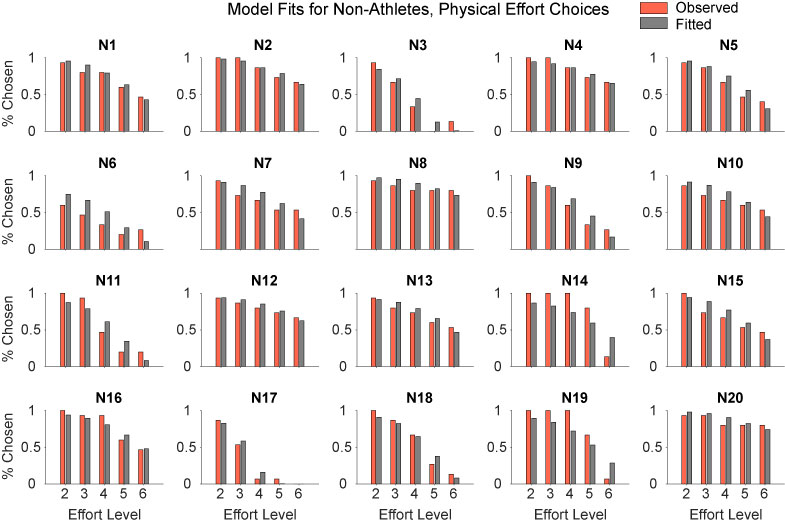
**

**Supplementary Figure 4.** Observed and model-predicted data are plotted for physical effort choices for each individual in the non-athlete group. Observed data are depicted in light red, and fitted data in grey.

**
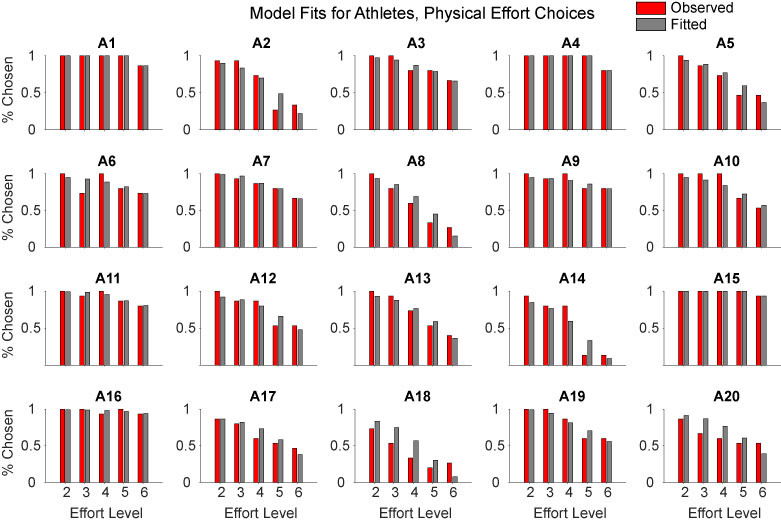
**

**Supplementary Figure 5.** Observed and model-predicted data are plotted for physical effort choices for each individual in the athlete group. Observed data are depicted in dark red, and fitted data in grey.

**Correlations between Physical and Cognitive Motivation**

We also asked whether there was a significant correlation between cognitive and physical effort discounting in the two groups (**Supplementary Figure 6**). Given that different functions best fitted cognitive effort discounting in athletes and non-athletes, the *k*-values for each individual in each group were first normalised ((*k* - μ_k_)/ σ_k_). We found that cognitive and physical effort *k*-values were significantly correlated across the entire cohort of athletes and non-athletes (Spearman’s *r* = 0.49, *p* < .005). This could suggest that the degree to which one is cognitively motivated correlates with one’s degree of physical motivation. However, because our effort discounting functions do not contain separate weights for reward and effort, an alternate interpretation is that this correlation may be driven simply by an individual’s overall sensitivity to reward.

*
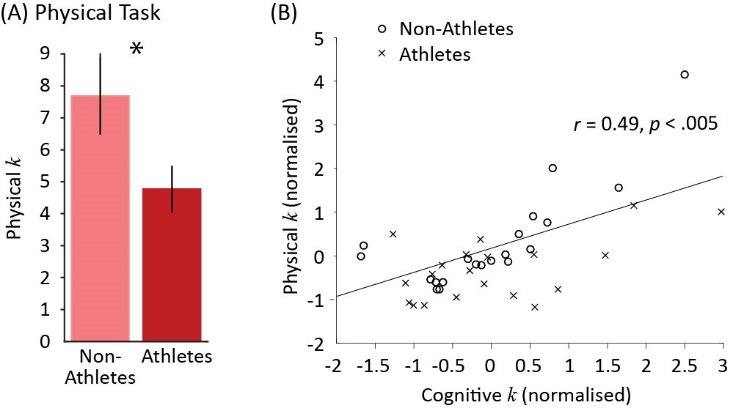
*

**Supplementary Figure 6.** Differences in cognitive and physical motivation between athletes and non-athletes. Motivation is indexed as subject-specific discounting parameters (*k*). (A) Athletes were more physically motivated than non-athletes. * *p* < .05. (B) Cognitive and physical effort discounting were significantly correlated across the whole group (o = non-athletes; x = athletes).

**Effect of Education on Cognitive Effort Discounting**

We note that there was no statistically significant difference in education between groups (*t*(38) = 1.70, *p* = .10). Nevertheless, to examine if there was an effect of education on choice preference, we correlated total years of education with *k_cog_* values. However, this correlation was not significant

(*r* = 0.09, *p* = .57). Nor were there significant correlations between total years of education and the gradient of the NASA mental demand scores for athletes (*r* = -0.12, *p* = .62) or non-athletes (*r* = 0.06, *p* = .79).

**Self-Reported Demand**

**Mental vs Physical Demand.** To confirm that participants' perception of effort increased with each increment of effort level in the corresponding domain, participants completed the NASA Task Load Index – a questionnaire used to assess subjective perceptions of task demand ^2^. **Supplementary Figure 7** shows the raw scores for the NASA task load index for mental and physical demand, plotted separately for the cognitive (A) and physical (B) effort tasks.

For the cognitive effort task, we conducted a three-way ANOVA on the factors of Group (athlete, non-athlete), Demand (mental, physical), and Effort (1-6). The main finding of relevance was a significant Group x Demand interaction (*F*(1,38) = 5.43, *p* = .03), which showed that physical demand scores were higher for non-athletes relative to athletes (non-athletes, -6.00 ± 0.66, vs athletes, -8.22 ± 8.23 ± 0.71, *p* = .03), but the mental demand scores were no different between groups (non-athletes 0.58 ± 0.66, vs athletes, 1.08 ± 0.66). Other significant findings were the main effects of Demand and Effort, which were involved in a significant interaction (Demand, *F*(1,38) = 184, *p* < .001; Effort, *F*(2.1, 80) = 103, *p* < .001; Demand x Effort, *F*(2.5, 95.4) = 463, *p* < .001). Decomposing this interaction revealed consistent increases in mental demand with increasing effort (*p*-values for all pairwise comparisons < .001), but there were less consistent differences in physical demand between consecutive levels of effort. Thus, the two main conclusions from this analysis are: (1) athletes found the cognitive effort task to be less physically demanding than non-athletes, and (2) the *cognitive effort task* resulted in significant increases in *mental demand* with increasing effort, and had less consistent effects on perceived physical demand.

The corresponding analysis for the physical effort task again revealed significant main effects of Demand and Effort, which were also involved in a significant interaction (Demand, *F*(1,38) = 43, *p* < .001; Effort, *F*(2.6, 98.4) = 180, *p* < .001; Demand x Effort, *F*(2.2, 83.7) = 44.0, *p* < .001). Decomposing this interaction revealed consistent increases in physical demand with increasing effort (*p*-values for all pairwise comparisons < .05), but less consistent differences in physical demand between consecutive levels of effort. This is a complementary finding to the preceding analysis on the cognitive effort task, showing that the *physical effort task* resulted in significant increases in *physical demand* with increasing effort, with less consistent effects on mental demand. Importantly, in this analysis, the main effect of Group and its interactions were not significant (all p-values > .23).

Together, these analyses show that both the cognitive and physical effort tasks resulted in overall greater perceived demand in the corresponding versus the alternate domain (i.e., difference between the solid and dashed lines in **Supplementary Figure 7**). Of note, the perceived demand in the alternate domain was always perceived as ‘low’ (i.e., below zero). In addition, the increasing effort levels of each task resulted in more consistent and significant increases in the perceived demand of the corresponding versus the alternate domain (i.e., steeper gradients of the solid versus the dashed lines). The only group difference was that athletes found the cognitive effort task to be less physically demanding than non-athletes. The reasons for this are unclear, although one possibility is that athletes’ physical training may result in them perceiving any task to be overall less physically demanding. Indeed, some sports psychologists have suggested that an important factor in motivating athletes to overcome effort costs is their lower perception of overall effort ^3,4^. We note, however, that this is a speculative interpretation.


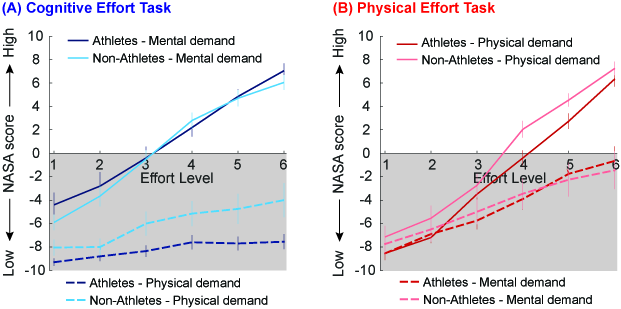


**Supplementary Figure 7.** Perceived mental and physical demand as indexed by the NASA Task Load Index. The NASA Task Load Index is a 21-point scale of perceived demand, from -10 (very low) to 10 (very high). Data are plotted separately for athletes and non-athletes in (A) the Cognitive Effort task, and (B) the Physical Effort task. Solid lines denote demand in the task-relevant domain (e.g., mental demand for the cognitive effort task), and dashed lines demand in the alternate domain. Athletes are in darker colours, and non-athletes in lighter colours. Perceived demand in the task-relevant domain progressively increased as a function of effort. In contrast, perceived demand in the alternate domain was always rated ‘low’ (grey-shading), and did not increase consistently as a function of effort. There were no statistical differences between the two groups, except that athletes found the cognitive effort task less physically demanding than non-athletes (panel A, dashed lines).

**Difference Scores.** We also asked whether the different cognitive effort discounting functions across the two groups was accompanied by perceived differences in relative cognitive demand. For each of the cognitive and physical effort tasks, we subtracted scores on the Physical from the Mental Demand subscales, in order to derive a metric indicating how much more mentally- vs physically-demanding each level was (positive = more mentally-demanding) (**Figure 5** of main text). Consistent with the above analyses, this metric showed that the cognitive and physical effort tasks are associated with greater increases in perceived demand in the corresponding domain. To determine whether the perceived demand curves differ between groups, we fit non-linear regression models to participants’ effort ratings using linear, parabolic and hyperbolic functions (using the *fitnlm* function in Matlab). The functions we used were:

Linear: $d=aE+b$

Parabolic: $d={aE}^{2}+b$

Hyperbolic: $d= \frac{a}{E}+b$

where *d* = perceived mental > physical demand (the ordinate on **Figure 5**); *a* is the gradient of the function; and *b* is a constant. We then compared the best fitting models with an AIC and BIC (**Supplementary Table 1**). Interestingly, this analysis resulted in similar conclusions to the main analyses on effort discounting functions. Specifically, parabolic functions described the pattern of perceived mental and physical demand for the corresponding tasks in athletes (**Figure 5B**). However, in non-athletes, a parabolic function described perceived physical demand, but a different function (in this case, linear) described perceived mental demand (**Figure 5A**). Together, these echo the pattern of results seen in the effort discounting functions, and are independent data indicating a difference in perceived cognitive effort between athletes and non-athletes.

Supplementary Table 1

*Results of model fits (AIC and BIC) for NASA demand scores in the cognitive and physical tasks. Shaded cells represent best-fitting models.*

|  | **AIC** | | | | **BIC** | | | |
| --- | --- | --- | --- | --- | --- | --- | --- | --- |
|  | **Non-Athletes** | | **Athletes** | | **Non-Athletes** | | **Athletes** | |
|  | **Cog** | **Phys** | **Cog** | **Phys** | **Cog** | **Phys** | **Cog** | **Phys** |
| *Linear* | **472** | 441 | 402 | 509 | **464** | 433 | 393 | 501 |
| *Parabolic* | 491 | **435** | **383** | **479** | 483 | **427** | **375** | **471** |
| *Hyperbolic* | 542 | 550 | 552 | 565 | 534 | 541 | 544 | 557 |

**References**

1 Klein-Flügge, M., Kennerley, S., Saraiva, A., Penny, W. & Bestmann, S. Behavioral modeling of human choices reveals dissociable effects of physical effort and temporal delay on reward devaluation. *PLoS Computational Biology* **11**, e1004116 (2015).

2 Hart, S. & Staveland, L. Development of NASA-TLX (Task Load Index): Results of empirical and theoretical research. *Advances in Psychology* **52**, 139-183 (1988).

3 Marcora, S. Counterpoint: afferent feedback from fatigued locomotor muscles is not an important determinant of endurance exercise performance. *Journal of Applied Physiology* **108**, 454-456 (2010).

4 Pageaux, B. The psychobiological model of endurance performance: an effort-based decision-making theory to explain self-paced endurance performance. *Sports Medicine* **44**, 1319-1320 (2014).
